# PLK1: A Promising Therapeutic Target for Prostate Cancer Treatment

**DOI:** 10.17161/sjm.v1i1.23189

**Published:** 2024

**Authors:** Jia Peng, Mansoureh Nouri, Hamed Maasoumyhaghighi, Jinghui Liu, Xiaoqi Liu

**Affiliations:** 1 Department of Toxicology and Cancer Biology, University of Kentucky, Lexington, Kentucky 40536, USA; 2 Markey Cancer Center, University of Kentucky, Lexington, Kentucky 40536, USA

**Keywords:** PLK1, prostate cancer, CRPC, PARPi, EZH2, treatment resistance

## Abstract

Prostate cancer is the most diagnosed cancer and a leading cause of cancer-related mortality among men in the United States. Androgen deprivation therapy (ADT) has long been the cornerstone of treatment for advanced prostate cancer. However, most patients eventually develop resistance, leading to progression into the lethal castration-resistant prostate cancer (CRPC) stage, which is associated with poor survival rates. While next-generation androgen receptor signaling inhibitors (ARSIs), such as enzalutamide and abiraterone, have revolutionized CRPC treatment, resistance to these therapies remains a significant challenge, rendering the disease incurable. Recent studies have identified Polo-like kinase 1 (PLK1), a master regulator of mitosis, as a key driver of prostate cancer progression, particularly in CRPC. PLK1 activation has been linked to critical pathways, including oxidative stress responses, lipid metabolism, and androgen receptor (AR) signaling, which collectively promote tumor growth and ARSI resistance. Moreover, PLK1 can regulate chromosomal stability and DNA damage repair pathways, contributing to sensitivity to irradiation therapy. Additionally, PLK1 plays a role in driving resistance to targeted therapies, such as PARP inhibitors, in BRCA-mutant CRPC. Compelling evidence further suggests that combining PLK1 inhibition with paclitaxel could serve as an effective therapeutic strategy to overcome resistance in CRPC by targeting microtubule dynamics. This review examines the mechanistic role and broader implications of PLK1 in CRPC treatment, offering valuable insights into potential combination therapies to improve efficacy and patient outcomes.

## Introduction

Prostate cancer is a significant health burden among men in the United States, with over 299,010 new cases projected in 2024, accounting for 29% of all newly diagnosed cancers in men. Additionally, it is estimated that 35,250 deaths will occur, making prostate cancer the second leading cause of cancer-related mortality in men, following lung and bronchus cancer[1]. Despite advancements in detection and treatment, prostate cancer outcomes remain highly dependent on the stage at diagnosis. Data from 2014 to 2020 indicate a 5-year relative survival rate of 97.5% for prostate cancer overall, with localized or regional cases achieving similarly high survival rates. However, the prognosis worsens dramatically for metastatic prostate cancer, where the 5-year survival rate drops to 36.6%[2]. Metastatic prostate cancer is not only incurable but also associated with significant morbidity and poor quality of life.

The prostate gland relies on androgens for its growth and function. Consequently, androgen deprivation therapy (ADT) remains the cornerstone of treatment for metastatic prostate cancer, providing substantial palliative benefits by reducing androgen levels and signaling[3]. However, the majority of patients eventually develop resistance to ADT, leading to disease progression and the transition to the lethal castration-resistant prostate cancer (CRPC) stage [4]. CRPC is characterized by the reactivation of androgen receptor (AR) signaling despite low circulating androgen levels, which drives tumor growth, recurrence, and metastasis[5].

At the CRPC stage, secondary AR-inhibitory therapies, including next-generation AR signaling inhibitors (ARSI) such as enzalutamide and abiraterone, are often used to achieve a more comprehensive blockade of androgen signaling[6]. ARSIs are initially effective for treating CRPC, but patients often eventually acquire resistance to continuous treatment with these agents[7].While ARSIs initially provide clinical benefits, most patients ultimately develop resistance to these agents. Resistance to ARSIs not only limits therapeutic options but also contributes to poor outcomes and increased mortality, posing a significant challenge to effective disease management [8]. The growing challenge underscores the urgent need for research into the underlying mechanisms driving ARSI resistance. This review explores the mechanistic role of PLK1 in CRPC and its therapeutic implications, highlighting its potential to overcome resistance to current treatments.

## Mechanisms Driving ARSI Resistance in CRPC

Multiple mechanisms drive resistance to ARSIs in prostate cancer. One such mechanism involves gene amplification, particularly of the AR gene, leading to its overexpression and enabling androgen-dependent growth despite low serum androgen levels and ARSI treatment[9]. Another mechanism is the presence of androgen receptor splice variants that encode truncated androgen receptors lacking the C-terminal ligand-binding domain but retaining the active N-terminal domain[10]. These splice variants act as constitutively active transcription factors, promoting gene activation independently of androgen binding. Additionally, the F876L missense mutation in the AR ligand-binding domain has been shown to confer resistance to second-generation antiandrogens, such as enzalutamide, in preclinical studies[11]. Detection of this mutation in plasma DNA from ARSI-treated patients with progressing disease suggests a role for next-generation antiandrogens paired with blood-based companion diagnostics to optimize treatment strategies.

Besides AR signaling, complex mechanisms that drive resistance to ARSIs have been extensively focused on in recent years. Through a combination of innovative approaches and rigorous analyses, key molecular pathways and therapeutic targets have been identified, offering new avenues for addressing this significant clinical challenge.

Investigations have revealed a pivotal role for the Wnt/β-catenin pathway in enzalutamide (ENZA) resistance. Bioinformatics analyses identified elevated levels of β-catenin and AR in resistant cells, which linked to reduced β-TrCP-mediated ubiquitination. Experimental activation of this pathway in ENZA-sensitive cells induced resistance, while its inhibition with the β-catenin inhibitor ICG001, combined with ENZA, synergistically suppressed tumor growth, stem-like marker expression, and cell proliferation. These findings underline the therapeutic potential of targeting the Wnt/β-catenin pathway in advanced prostate cancer[12]. Further studies have highlighted the significant involvement of noncanonical Wnt signaling in ENZA resistance. Elevated expression of Wnt5a, RhoA, and ROCK was observed in resistant cells compared to their sensitive counterparts. Studies have shown that inhibiting ROCK using Y27632 or genetic knockdown resensitized resistant cells to ENZA, disrupted epithelial-mesenchymal transition (EMT), and reduced cell invasion and migration. These effects were further validated in xenograft models, where the combination of Y27632 and ENZA synergistically suppressed tumor growth[13].

Another key finding identified glutathione S-transferase Mu 2 (GSTM2) as a critical determinant of resistance to second-generation AR inhibitors (SG-ARIs). Elevated GSTM2 expression, driven by the aryl hydrocarbon receptor (AhR), enabled resistant cells to mitigate oxidative stress and suppress the p38 MAPK pathway. Overexpression of GSTM2 in ENZA-sensitive cells conferred resistance not only to ENZA but also to apalutamide (APA) and darolutamide (DARO). These results point to GSTM2 as an actionable target for overcoming SG-ARI resistance[14]. Through RNA sequencing and bioinformatics analyses, NOTCH signaling was identified as a key pathway driving ENZA resistance. Resistant cells displayed elevated levels of cleaved NOTCH1, HES1, and c-MYC, which correlated with AR expression. Inhibiting ADAM10, a regulator of NOTCH cleavage, or directly targeting NOTCH1 reduced proliferation and resensitized cells to ENZA. These results were further supported by xenograft studies where combining the NOTCH inhibitor PF-03084014 with ENZA significantly suppressed tumor growth by enhancing apoptosis and reducing proliferation[15].

The role of DNA methylation in ENZA resistance has also been highlighted by the finding that DNA methyltransferase (DNMT) activity, particularly DNMT3B expression, was upregulated in resistant cells. Overexpression of the DNMT3B variant DNMT3B3 promoted resistance, while DNMT3B knockdown or treatment with the DNA methylation inhibitor decitabine resensitized cells to ENZA. Mechanistic studies revealed that decitabine reduced AR-V7 expression and modulated genes related to apoptosis and DNA repair, resulting in enhanced tumor suppression in xenografts when combined with ENZA[16]. The role of epigenetic regulation in ARSI resistance was underscored by the findings on the enhancer of zeste homolog 2 (EZH2). It was demonstrated that EZH2 directly suppresses AR-dependent transcription, contributing to ENZA resistance. Inhibiting EZH2 using GSK126 synergistically enhanced ENZA efficacy by promoting apoptosis and reducing proliferation in resistant models. Further analyses revealed a strong correlation between EZH2 and AR expression in clinical datasets, positioning EZH2 inhibition as a promising therapeutic strategy in CRPC[17].

A crucial discovery was the identification of casein kinase 1α (CK1α) as a critical modulator of ENZA resistance. By conducting a kinome-wide CRISPR-Cas9 knockout screen, it was demonstrated that CK1α phosphorylates ATM at serine residue S1270, stabilizing this vital initiator of DNA double-strand break (DSB) repair. In ENZA-resistant cell lines and patient-derived xenografts, CK1α inhibition successfully restored DSB signaling, leading to enhanced apoptosis and growth arrest. This finding highlights the therapeutic potential of CK1α inhibition in combating ARSI resistance[18]. The role of cholesterol biosynthesis in ENZA resistance has been explored, revealing that 3-hydroxy-3-methyl-glutaryl-CoA reductase (HMGCR) expression is elevated in resistant cells. This elevation is associated with androgen receptor (AR) stabilization through mTOR activation. Statins, including simvastatin, effectively reduced HMGCR and AR levels, resensitizing cells to ENZA. Combination treatment with simvastatin and ENZA demonstrated significant tumor suppression in cell culture models and xenograft models, suggesting that targeting cholesterol biosynthesis could be a viable approach to overcoming ARSI resistance[19]. These findings collectively provide a comprehensive understanding of the molecular mechanisms driving ARSI resistance and offer actionable insights for developing more effective combination therapies in CRPC.

## The Role of PLK1 in Sensitizing Cancer Therapies for CRPC

Polo-like kinase 1 (PLK1) is a serine/threonine kinase that plays a pivotal role in cell cycle regulation, particularly during mitosis[20].It is a master regulator of several key processes required for proper cell division, including initiating mitotic entry [21, 22], assembling the mitotic spindle [23, 24], promoting centrosome maturation [25-27], nuclear envelope breakdown (NEBD)[28, 29], regulating kinetochore activity [30, 31], chromosome condensation and segregation[32, 33], activating the anaphase-promoting complex/cyclosome (APC/C)[34, 35], and coordinating cytokinesis[36, 37]. Given its central role in cell division and maintaining genomic stability, PLK1 has become a focal point in both basic and clinical cancer research. Overexpression of PLK1 has been observed in various human cancers, including prostate cancer, where it is associated with enhanced cellular proliferation and poor prognosis[38-41]. This highlights the critical need for further investigation into the mechanisms by which PLK1 contributes to the initiation and progression of prostate cancer.

**Figure 1.**
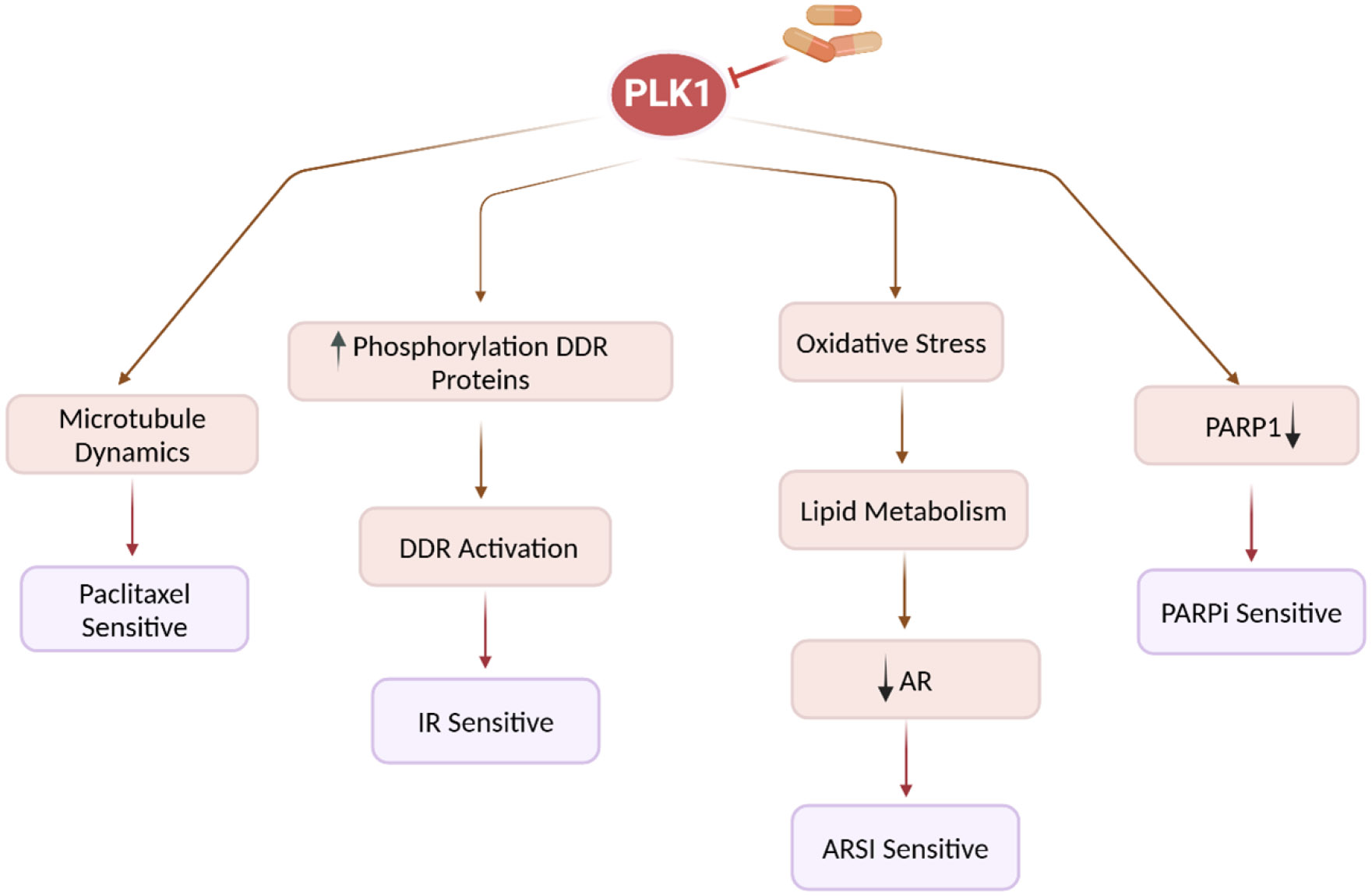
Plk1 Inhibition Enhances Therapeutic Sensitivity in Prostate Cancer. PLK1 inhibition enhances responses to ARSIs, PARP inhibitors, paclitaxel, and IR treatment by targeting metabolic pathways, microtubule dynamics, PARP1 activity, and DNA damage repair (DDR) mechanisms, effectively overcoming therapeutic resistance in prostate cancer.

Advances in high-throughput genetic technologies, such as RNA sequencing and CRISPR screens, along with studies in animal models and human prostate cancer tissues, have facilitated detailed analyses of molecular alterations driving prostate cancer initiation and progression [42]. These efforts have highlighted PLK1 as a critical regulator of mitotic cell division, particularly under conditions of replicative stress and chromosomal instability. In prostate epithelial cells, elevated PLK1 levels promote malignant transformation, enhanced cell migration, and epithelial-to-mesenchymal transition, which are correlated with higher disease grades [43]. Additionally, PLK1 inhibits the proapoptotic activity of the FOXO1 transcription factor[44], regulating BRD4and DNMT3a stability[45, 46], and interacting with the Wnt/β-catenin pathway in CRPC, making it a promising target for therapeutic intervention[47]. Given its multifaceted role in CRPC progression, PLK1 has emerged as a potential therapeutic target to sensitize cancer treatments and overcome resistance. The following sections explore the diverse therapeutic strategies involving PLK1 inhibition, including its ability to enhance responses to ARSIs, ionizing radiation therapy, PARP inhibitors, and paclitaxel, as summarized in Figure 1.

## Enhancing Sensitivity to ARSI Treatments

Prostate cancer progression is driven by oxidative stress, AR signaling, and activation of the PI3K-AKT-mTOR pathway. Notably, Plk1 signaling and lipid metabolism have been identified as the most significantly upregulated pathways in prostate cancer[48]. Studies reveal that oxidative stress activates both the PI3K–AKT–mTOR pathway and AR signaling in a Plk1-dependent manner in prostate cells[49, 50]. Pharmacological inhibition of the PI3K–AKT–mTOR pathway effectively abrogated oxidative stress-induced activation of AR signaling. Additionally, Plk1 modulation influenced cholesteryl ester accumulation via the SREBP pathway. Importantly, Plk1 inhibition enhanced the therapeutic response to ARSIs and overcame resistance to these agents. These findings underscore the role of Plk1 activation in coordinating oxidative stress responses, lipid metabolism, and AR signaling to sustain prostate cancer progression. These results provide a strong mechanistic rationale for exploring Plk1 inhibitors in combination with ARSIs to improve therapeutic outcomes in CRPC[51]. Another study showed that the microtubule-stabilizing drug inhibits AR signaling by preventing its nuclear accumulation, a process dependent on microtubule stabilization. In this context, PLK1 plays a critical role by suppressing kinetochore-microtubule dynamics, thereby stabilizing initial attachments during prometaphase. This mechanism highlights how PLK1-associated kinase activity contributes to the constitutive activation of AR signaling in CRPC. Supporting these findings, a recent study demonstrated that Plk1 inhibition synergizes with the ARSI abiraterone by inducing mitotic defects in ARSI-resistant metastatic CRPC[52]. Furthermore, another study reported that co-targeting Plk1 and the androgen receptor significantly improves therapeutic sensitivity in paclitaxel-resistant prostate cancer[53].

## Combination with Ionizing Radiation Therapy

Using a conditional expression system, Plk1 transgenic mouse models were developed to investigate the role of Plk1 in tumorigenesis. Overexpression of Plk1 in mouse embryonic fibroblasts derived from these transgenic mice resulted in aberrant mitosis, leading to chromosomal instability characterized by aneuploidy and apoptosis. Despite these cellular abnormalities, Plk1 overexpression alone did not result in any overt phenotypic changes or malignant tumor formation in the mice, even over extended periods, suggesting it may act as a passenger rather than a driver. This observation suggests that additional oncogenic factors are required for tumor development in Plk1-overexpressing mice. Given Plk1’s involvement in regulating the DNA damage response (DDR) pathway, the Plk1-overexpressing mice were exposed to ionizing radiation (IR). These mice displayed significantly heightened sensitivity to IR compared to their wild-type counterparts. Tumorigenesis analysis revealed a substantial reduction in latency for tumor development following IR exposure in Plk1-overexpressing mice. Mechanistically, Plk1 overexpression was associated with diminished phosphorylation of key DDR proteins, including ATM, Chk2, and histone H2AX, after IR treatment. Additionally, gene expression analysis suggested that elevated Plk1 levels downregulate several critical DDR genes. These findings indicate that Plk1 can regulate tumorigenesis by influencing chromosomal stability and DDR pathways[54]. As such, targeting Plk1 in combination with ionizing radiation may represent a promising therapeutic strategy to enhance treatment efficacy in prostate cancer.

## Overcoming PARP Inhibitor Resistance

Olaparib, an FDA-approved PARP inhibitor (PARPi), shows considerable promise as a synthetic lethal therapy for BRCA-mutant CRPC. However, growing evidence suggests that BRCA-mutant cells can acquire resistance to PARPi, with the underlying mechanisms remaining poorly defined. It has been demonstrated that combining olaparib with the Plk1 inhibitor significantly enhances the sensitivity of BRCA1-deficient CRPC cells and CRPC xenograft tumors to PARPi[55]. Mechanistically, olaparib monotherapy bypasses the G1–S checkpoint, leading to increased Plk1 expression, which attenuates its therapeutic efficacy. Notably, in BRCA1 wild-type C4-2 cells, Plk1 inhibition also improves olaparib’s effectiveness, even in the presence of mutant p53. These findings underscore the critical role of Plk1 in driving PARPi resistance in BRCA-mutant CRPC and suggest that targeting Plk1 may expand the therapeutic scope of PARP inhibitors, potentially benefiting non-BRCA-mutant patient populations.

## Sensitize Taxane-based Chemotherapies

Taxane-based chemotherapy remains the standard treatment for CRPC, yet resistance to this therapy inevitably emerges in nearly all patients. Studies reveal that paclitaxel exerts its anti-tumor effects in CRPC by inhibiting AR activity through disruption of microtubule dynamics. Research has demonstrated that Plk1 phosphorylates CLIP-170 and p150Glued, two key regulators of microtubule dynamics[56]. Plk1-mediated phosphorylation of these proteins influences cellular sensitivity to paclitaxel. Notably, expression of non-phosphorylatable mutants of CLIP-170 and p150Glued enhances paclitaxel-induced apoptosis, promotes AR protein degradation, and reduces nuclear AR accumulation in response to androgen stimulation in prostate cancer cells[57]. Moreover, cells expressing non-phosphorylatable CLIP-170 mutants exhibit impaired microtubule dynamics, providing mechanistic insights into the role of Plk1 kinase activity in sustaining AR signaling in CRPC. These findings suggest that combining Plk1 inhibition with paclitaxel holds significant potential as a therapeutic strategy to overcome resistance in CRPC. Supporting this, another study demonstrated that combining Plk1 inhibition with nocodazole, a microtubule-targeting agent with a mechanism similar to taxanes, produced synergistic antitumor effects in vitro while sparing normal prostate epithelial cells, further emphasizing the promise of targeting Plk1 in microtubule-associated therapies for CRPC[44].

## PLK1 Inhibitors: Clinical Progress and Therapeutic Potential in CRPC

PLK1 has become a prominent therapeutic target due to its critical role in cell cycle regulation and tumor progression. Numerous PLK1 inhibitors have been developed and evaluated in both preclinical and clinical settings, showing promising results across various cancers, including CRPC. This section provides an overview of the clinical progress of several PLK1 inhibitors and explores their therapeutic potential in the treatment of CRPC.

### 1. BI2536

BI 2536 is one of the earliest ATP-competitive inhibitors developed to target PLK1, a critical regulator of mitotic progression and cell cycle control. As a dihydropteridinone derivative, BI 2536 effectively inhibits PLK1 activity at nanomolar concentrations, inducing G2/M phase arrest and mitotic abnormalities such as monopolar spindle formation. This disruption leads to apoptosis and tumor cell death, making it a potent anticancer agent in preclinical models[40, 58, 59]. BI-2536 was the first PLK1 inhibitor to enter clinical trials, there are 11 clinical trials about BI2536 posted on ClinicalTrials.gov. However, dose-limiting toxicities, particularly reversible neutropenia, posed challenges for its widespread clinical application in monotherapy[60-62].

In the context of prostate cancer, BI2536 shows promise in CRPC treatment. It acts synergistically with other therapies, such as PARP inhibitors, and metformin to enhance antitumor effects[55, 63]. By inhibiting PLK1, BI2536 induces cell cycle arrest, and apoptosis, and increases sensitivity to other treatments. It affects key pathways like PI3K–AKT–mTOR, Wnt/β-catenin, and androgen receptor signaling[64-66]. Preclinical studies in CRPC models demonstrate significant tumor growth inhibition and enhanced efficacy when combined with other treatments[67]. BI2536 offers a potential new approach to managing CRPC by augmenting the effectiveness of existing therapies.

### 2. Volasertib (BI6727)

Volasertib (BI6727), an advanced ATP-competitive inhibitor of PLK1, was developed by Boehringer Ingelheim as a refined successor to BI2536[68]. Volasertib induces G2/M cell cycle arrest and apoptosis in a variety of tumor cell lines. It has demonstrated strong antitumor activity in preclinical models, including those of acute myeloid leukemia (AML), non-small cell lung cancer (NSCLC), and ovarian cancer, among others[69, 70]. Volasertib boasts improved pharmacokinetic properties compared to its predecessor BI2536, such as better tissue penetration and a longer half-life[71]. Clinical trials highlighted its manageable toxicity profile, with reversible hematologic side effects like neutropenia and thrombocytopenia at the maximum tolerated doses[70]. Although monotherapy yielded only modest results, Volasertib showed promise in combination therapies, such us with low-dose cytarabine in elderly AML and combined with Afatinib in advanced solid tumors patients, significantly improving response rates and overall survival[72, 73].

Volasertib has shown promise in the treatment of CRPC by targeting PLK1, a critical regulator of mitosis often overexpressed in CRPC. Preclinical studies indicate that Volasertib induces G2/M arrest and apoptosis which is crucial in CRPC cell[67, 74]. Moreover, one study has demonstrated that combining volasertib with docetaxel (DTX), a standard chemotherapy for CRPC, results in strong synergistic effects. The combination of volasertib and DTX caused greater inhibition of cell viability compared to either drug alone in CRPC cell lines[75]. While studies specific to CRPC are limited, these findings suggest that Volasertib could be integrated into combination therapies to improve outcomes in resistant prostate cancer.

### 3. Onvansertib (NMS-1286937, NMS-P937, PCM-075)

Onvansertib is a third-generation, ATP-competitive inhibitor of PLK1 developed by Nerviano Medical Science[76, 77]. It potently inhibits PLK1 kinase activity in biochemical assays, with an IC50 value of 2 nmol/L. Onvansertib also demonstrates high potency in numerous cell lines, with IC50 values below 100 nmol/L in 60 out of 137 tested cell lines. Its mechanism of action involves inducing mitotic cell-cycle arrest followed by apoptosis, a process verified in a mouse xenograft model, which confirmed a clear PLK1-related mechanism[78].

Onvansertib is orally bioavailable, with a human half-life of approximately 24 hours, and is well tolerated in patients with AML[79]. In a Phase I trial, its manageable safety profile was established, with thrombocytopenia and neutropenia identified as primary dose-limiting toxicities[80]. The compound has been evaluated in multiple clinical trials across various cancer types, including AML, metastatic colorectal cancer (mCRC) with KRAS mutations, and CRPC, showing promising results in combination therapies. For AML, a Phase Ib study demonstrated that the combination of Onvansertib and decitabine was well tolerated and exhibited antileukemic activity, particularly in patients showing target engagement and decreased mutant circulating tumor DNA (ctDNA) post-treatment. This combination is being further explored in an ongoing Phase II trial[79]. For mCRC, a Phase Ib study showed that Onvansertib combined with FOLFIRI/bevacizumab was well tolerated and exhibited promising efficacy as a second-line treatment for KRAS-mutant mCRC[81]. A subsequent Phase II trial confirmed significant activity of this combination, particularly in patients without prior bevacizumab exposure[82]. These findings lead to its evaluation in the first-line setting (NCT06106308). Additional ongoing trials are investigating Onvansertib in recurrent or refractory chronic myelomonocytic leukemia (NCT05549661), relapsed small cell lung cancer (NCT05450965), triple-negative breast cancer (TNBC) with paclitaxel (NCT05383196), and pancreatic cancer (NCT04005690), underscoring its broad therapeutic potential.

In CRPC, Onvansertib is being investigated for its ability to enhance efficacy and overcome resistance to ARSIs. Clinical trials have evaluated its combination with abiraterone and prednisone in patients with metastatic CRPC who progressed on abiraterone (NCT03414034). Preclinical studies further support these efforts, demonstrating that Onvansertib synergizes with abiraterone in vitro and in vivo to target a subset of androgen-independent cancer cells[83]. Taken together, these findings position Onvansertib as a promising therapeutic option to overcome ARSI resistance in CRPC.

### 4. GSK461364

GSK461364, a selective ATP-competitive PLK1 inhibitor developed by GlaxoSmithKline, is a thiophene amide that effectively induces G2/M cell cycle arrest and apoptosis in cancer cells[84]. With a Ki*app of <0.5 ± 0.1 nmol/L, GSK461364 exhibits at least 1,000-fold selectivity for PLK1 compared to other kinases, including PLK2 and PLK3. It inhibits cell growth in 89% of 74 tested cancer cell lines, achieving a GI50 ≤ 100 nmol/L after 72 hours of drug exposure[84]. Preclinical studies have demonstrated its antitumor activity, including tumor growth inhibition, prevention of brain metastases in breast cancer models, and induction of mitotic arrest and apoptosis in osteosarcoma[85-87]. Additionally, it suppresses gastric cancer cell proliferation and induces cellular senescence. In phase I clinical trials for solid tumors, dose-limiting toxicities included venous thrombotic emboli, neutropenia, and sepsis, leading to a recommended phase II dose of 225 mg, combined with anticoagulants to mitigate adverse effects[88].

In prostate cancer, GSK461364 has demonstrated potential in combination therapies. A study combining GSK461364 with the BRD4 inhibitor JQ1 revealed strong synergistic effects in CRPC cell lines and xenograft models. This synergy is attributed to two mechanisms: PLK1 inhibition elevates c-MYC levels, which is directly counteracted by JQ1, and the combined inhibition of PLK1 and BRD4 synergistically disrupts AR signaling[66]. These findings highlight the promise of using GSK461364 in combination therapies for CRPC treatment.

Several other PLK1 inhibitors have been evaluated in clinical trials, including TAK-960, CFI-400945, Rigosertib (ON-01910), CYC140, and BAL0891, with the latter three still undergoing active investigation. Among these, the non-ATP-competitive inhibitor Rigosertib has been extensively studied, with four clinical trials reaching phase III for myelodysplastic syndromes (MDS) and metastatic pancreatic cancer. While research on these inhibitors in prostate cancer is limited, the critical role of PLK1 in CRPC suggests their potential utility, particularly as part of combination therapies. Further exploration of these agents could offer promising new strategies for CRPC treatment.

The FDA has granted orphan drug status and breakthrough therapy to some PLK1 inhibitors, including volasertib and onvansertib, also volasertib and Rigosertib have reached phase III, however no PLK1 inhibitor has yet been granted marketing approval by the FDA, indicating that challenges remain. One of the primary challenges is the complexity of selectively targeting PLK1 while minimizing off-target effects on other kinases, which can lead to toxicity. Despite the high specificity of some inhibitors, dose-limiting toxicities such as neutropenia, thrombocytopenia, and venous thromboembolism frequently occur in clinical trials[60-62, 70, 80]. Cancer cells often develop resistance to PLK1 inhibitors, particularly through mutations at the ATP-binding region of the PLK1 kinase domain. This resistance can significantly reduce the efficacy of these inhibitors in clinical settings[67, 89]. Combination therapies involving PLK1 inhibitors and other agents such as chemotherapy or immunotherapy have shown superior effects in preclinical models, promoting apoptosis, disrupting the cell cycle, and overcoming resistance compared to monotherapies[67]. While early clinical trials have shown promise, some combinations have failed to improve patient survival. This highlights the need for further research to identify and validate novel co-targets that can optimize PLK1-based combination therapies.

## Summary

PLK1 has emerged as a pivotal therapeutic target in prostate cancer, particularly in addressing resistance in CRPC. This review highlights PLK1’s multifaceted role in cell cycle regulation, genomic stability, and its involvement in key pathways such as oxidative stress response, lipid metabolism, and DNA damage repair. Elevated PLK1 levels contribute to tumor initiation following ionizing radiation and drive tumor progression and therapeutic resistance, particularly against ARSIs, PARP inhibitors, and taxane-based chemotherapies like paclitaxel. Evidence underscores the therapeutic potential of PLK1 inhibitors, especially when combined with existing treatments, to improve outcomes in CRPC. This paper provides comprehensive insights into PLK1’s mechanisms and its promising role in future therapeutic strategies for prostate cancer.
